# Profound Morphological Changes in the Erythrocytes and Fibrin Networks of Patients with Hemochromatosis or with Hyperferritinemia, and Their Normalization by Iron Chelators and Other Agents

**DOI:** 10.1371/journal.pone.0085271

**Published:** 2014-01-09

**Authors:** Etheresia Pretorius, Janette Bester, Natasha Vermeulen, Boguslaw Lipinski, George S. Gericke, Douglas B. Kell

**Affiliations:** 1 Department of Physiology, University of Pretoria, Arcadia, South Africa; 2 Joslin Diabetes Center, Harvard Medical School, Boston, Massachusetts, United States of America; 3 AMPATH National Reference Laboratory, Centurion, South Africa; 4 School of Chemistry and The Manchester Institute of Biotechnology, The University of Manchester, Lancs, United Kingdom; National University of Singapore, Singapore

## Abstract

It is well-known that individuals with increased iron levels are more prone to thrombotic diseases, mainly due to the presence of unliganded iron, and thereby the increased production of hydroxyl radicals. It is also known that erythrocytes (RBCs) may play an important role during thrombotic events. Therefore the purpose of the current study was to assess whether RBCs had an altered morphology in individuals with hereditary hemochromatosis (HH), as well as some who displayed hyperferritinemia (HF). Using scanning electron microscopy, we also assessed means by which the RBC and fibrin morphology might be normalized. An important objective was to test the hypothesis that the altered RBC morphology was due to the presence of excess unliganded iron by removing it through chelation. Very striking differences were observed, in that the erythrocytes from HH and HF individuals were distorted and had a much greater axial ratio compared to that accompanying the discoid appearance seen in the normal samples. The response to thrombin, and the appearance of a platelet-rich plasma smear, were also markedly different. These differences could largely be reversed by the iron chelator desferal and to some degree by the iron chelator clioquinol, or by the free radical trapping agents salicylate or selenite (that may themselves also be iron chelators). These findings are consistent with the view that the aberrant morphology of the HH and HF erythrocytes is caused, at least in part, by unliganded (‘free’) iron, whether derived directly via raised ferritin levels or otherwise, and that lowering it or affecting the consequences of its action may be of therapeutic benefit. The findings also bear on the question of the extent to which accepting blood donations from HH individuals may be desirable or otherwise.

## Introduction

Iron overload is associated with many pathological conditions, including liver and heart disease, neurodegenerative disorders, diabetes, hormonal abnormalities immune system abnormalities, heart failure, and in particular in the more classical conditions recognised as ‘iron overload’ diseases such as hemochromatosis (e.g. [1]–[14]). Moderate iron loading is also known to accelerate thrombus formation after arterial injury, to increase vascular oxidative stress, and to impair vasoreactivity [5], [15]–[17]. Furthermore, iron-induced vascular dysfunction may contribute to the increased incidence of ischemic cardiovascular events that have been associated with chronic iron overload [15], [18]–[20]. Poorly liganded iron is the main culprit, and plays a fundamental role in the development of pathology [1], [2], while copper dysregulation may also be of significance [21].

In 1976, Simon and co-workers first noted that idiopathic hemochromatosis is a genetic disease and suggested that the gene(s) responsible for the disease may be linked to the histocompatibility genes [22]. The relevant (*HFE*) gene discovery was only reported in 1996 [23]. HH is now probably the most well-known genetic iron overload disease [24]–[26]. The most common types of HH are caused by a C282Y or H63D mutation in the protein encoded by the *HFE* gene [27]–[38]; HH individuals may also be C282Y/H63D or present as a variety of heterozygotes, where they have one copy of each of the mutation and a wild type copy [39], [40]. These types of HH are called type 1 (classical *HFE* gene mutations, resulting in a cysteine-to-tyrosine substitution at amino acid 282 - C282Y) or a histidine-to-aspartate substitution at amino acid 63 - H63D [41]. However, there are also non-*HFE* haemochromatoses, which include all hemochromatosis disorders that are unrelated to the typical *HFE* mutations [42]. Mutations in different genes are responsible for the distinct types of non-classical *HFE* hemochromatosis, including hepcidin [43], [44] and hemojuvelin (type 2 or juvenile hemochromatosis - resulting from mutations in iron regulatory protein, hemojuvelin – HJV gene [45]), transferrin receptor 2 (type 3 hemochromatosis -TFR2 gene [46], [47]; and mutations in the iron exporter, ferroportin 1 [48] (mutated in type 4, the atypical dominant form of primary iron overload - SLC40A1 gene) [41], [42], [49].

In the current work, HH will refer to type 1 or classical hemochromatosis, based on mutations in the *HFE* gene, as confirmed herein by genotyping. The genes linked to HH cause disruption of the mechanisms that regulate iron absorption, leading to progressive increase of total body iron and organ damage [42]. Therefore, HH is indicative of disruption of the *HFE* gene product, as well as commonly (but not inevitably) a persistent elevation of serum ferritin concentration [42], [50]. Here we also classify hyperferritinemia (HF) as occurring in individuals with high serum ferritin levels (higher than 200 ng/mL^−1^ for females and 300 ng/mL^−1^ for males) but not with the genetic mutation in the *HFE* gene (these individuals were tested for all combinations of the C282Y, H63D as well as S65C mutations, and found to be wild type for these mutations). Serum ferritin levels remain an important indication of iron overload in HH, and therefore is an important diagnostic tool [41], [51]–[53].

One of the main medical complications of hemochromatosis is that uncontrolled iron leads to tissue damage derived from free radical toxicity caused by the excessive levels of this metal [41], [54]–[57]. Previously, we have shown that when ferric iron is added to whole blood taken from healthy individuals, the red blood cell morphology is changed [58]. We have also seen that in diabetes (where iron overload is sometimes also present - and these diseases may be mutually causative [2], [13], [51], [59]–[65]) RBCs are elongated and twist around fibrin fibers [66]. The possible hydroxyl radical formation, due to excess iron, or the excess iron itself, may therefore, as can many drugs [67], change red blood cell and fibrin fiber ultrastructure, as both RBCs and coagulation factors are exposed to particularly high serum ferritin and/or iron that may ultimately cause hydroxyl radicals to be produced in the serum.

**Figure 1:**
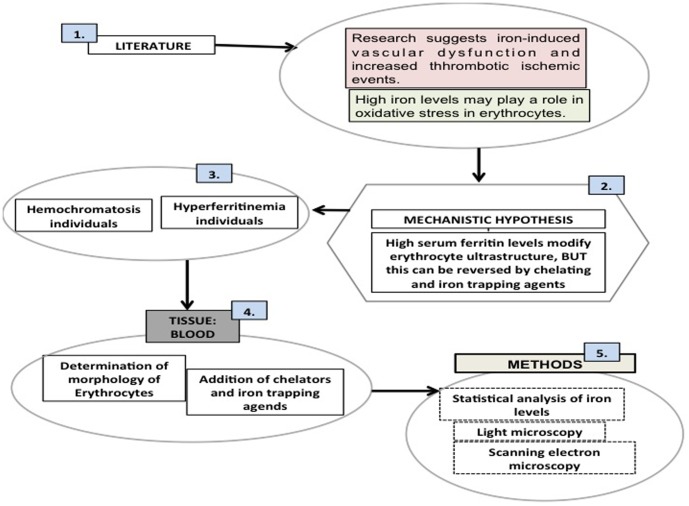
An overview figure summarizing the contents of this manuscript. **1**) Literature suggests that there is a rationale for looking at the vascular system and particularly blood; **2**) we propose a mechanistic hypothesis; **3**) our sample was chosen to be a random group of hereditary hemochromatosis and hyperferritinemia individuals, along with controls; **4**) our tissue of choice was blood where we looked at the morphology of erythrocytes with and without the addition of chelators and iron trapping compounds; **5**) our choice of methods involved microscopy techniques as well as statistical analysis of iron levels.

In view of the above, we here investigate the RBC morphology in blood from individuals with hemochromatosis, in the presence or absence of thrombin, and also study fibrin fiber morphology. To make the logic behind this study and its mechanistic hypotheses the clearer, and following the precepts of Wong [68], we now include a descriptive Figure 1 that sets out the structure of the manuscript.

We also add iron-chelating and other compounds to determine any stabilizing effect of these substances on both RBC ultrastructure and fibrin fiber morphology. We observe remarkable difference in morphology between RBCs from hemochromatosis and hyperferritinemic individuals relative to those from normal controls, with certain kinds of iron chelator and hydroxyl radical scavenger being able to return the aberrant morphology to near-normal states. It is unknown as to whether this aberrant morphology of itself contributes to disease pathology (in the way that aberrant morphology is well known to do in the case of sickle cell disease [69]), but the present findings would possibly open the debate about the desirability of using blood donations from HH individuals.

## Materials and Methods

Ethical approval was granted at the University of Pretoria (HUMAN ETHICS COMMITTEE: FACULTY OF HEALTH SCIENCES) under the name E Pretorius. Healthy individuals: written informed consent was obtained from all healthy individuals used as controls. Hemochromatosis and wild type blood: throwaway blood was obtained from a routine genetics laboratory (AMPATH National Reference Laboratory) after blood was sent for genetic testing. The blood tubes came from all over South Africa from various Pathology depots, to a central Genetic laboratory for genetic testing. The Genetics laboratory obtained written consent in order to perform the genetic testing. As for the erythrocyte analysis, no additional written consent was obtained, as the individuals were not identified for the academic part of the study. No contact details were available where patients could be identified or contacted. Given this, the institutional review board waived the need for written informed consent, for the academic part of the study, from these participants.

Twenty non-smoking healthy individuals with no chronic diseases and who do not use any medication, served as control subjects. SEM pictures of their RBC were compared to those in our SEM database (of thousands of micrographs) and found to be comparable. Hemochromatosis individuals were previously genotyped as H63D/H63D; C282Y/C282Y; H63D/wild type and C282Y/wild type and all where Cacausians. Furthermore, wild types, with high serum ferritin levels were included in the current study. Blood samples from Hemochromatosis individuals were obtained from the South African National Blood Services. Serum ferritin levels, free iron, transferrin and % saturation were also measured. Currently a hemochromatosis SEM database of RBC and fibrin fiber networks with and without thrombin is being created. Ethical clearance was obtained for the study from the University of Pretoria Human Ethics Committee: E Pretorius as principal investigator (Ethics Approval Number 151/2006).

Genomic DNA from 5 ml human blood samples collected in EDTA tubes was isolated using the MagneSil KF Genomic System on the KingFisher ML instrument. A multiplex PCR to determine the *HFE* C282Y and H63D mutations was performed using fluorescent hybridization probes specifically adapted for PCR in glass capillaries using the ROCHE 480 Light Cycler. A Melting Curve program is used to genotype the human genomic DNA samples. The resulting melting peaks allow discrimination between the homozygous (wild type or mutant) as well as the heterozygous genotype [70].

Whole blood samples from healthy individuals and individuals with hemochromatosis were obtained in Ethylenediaminetetraacetic acid (EDTA) blood tubes [71]. To prepare whole blood smears, 10 µl aliquots were directly placed on a glass cover slip with and without the addition of 5 µl human thrombin (20 U/mL). Platelet rich plasma (PRP) (10 µl aliquots) was also prepared and mixed with 5 µl thrombin [8].

Desferal, salicylate, sodium selenite and clioquinol were prepared as stock solutions at 10 mM concentrations (stock solution 1) as well as a lower stock solution of 0.5 mM (stock solution 2) for each of the compounds used. The final concentrations after dilution and after adding stock solution 1 and 2, are shown in Table 1.

**Table 1:** Volumes of platelet rich plasms (PRP), whole blood (WB) and thrombin (T) versus concentration and volume of iron-chelating and related compound.

Volumes and concentrations	Final compound concentration
10 µl PRP+5 µl T+5 µl of 10 mM (stock solution 1) compound.	2.5 mM
10 µl WB+5 µl T+5 µl of 10 mM (stock solution 1) compound.	2.5 mM
10 µl PRP+5 µl T+5 µl of 0.5 mM (stock solution 2) compound.	0.125 mM
10 µl WB+5 µl T+5 µl of 0.5 mM (stock solution 2) compound.	0.125 mM
10 µl WB+5 µl of 10 mM (stock solution 1) compound.	3.33 mM
10 µl WB+5 µl of 0.5 mM (stock solution 2) compound.	0.167 mM

Whole blood smears for light microscopy were prepared by making a blood smear with 10 µl of whole blood on to a microscope slide. The smear was allowed to dry on a hotplate for 5 minutes or until completely dry followed by fixing in 100% methanol and staining with methylene blue for (4 minutes) and eosin (30 seconds). Smears were viewed with a Nikon Optiphod transmitted light microscope (Nikon Instech Co., Kanagawa, Japan).

Axial ratios of RBCs from healthy individuals, from those with HH mutations, and from various wild types with or without chelating and hydroxyl trapping compounds (0.5 mM concentration), were captured using ImageJ (ImageJ is a public domain, Java-based image processing program developed at the National Institutes of Health: http://rsbweb.nih.gov/ij/). Axial ratios were always greater than (or equal to) 1 by using the largest diameter overall as the numerator and the largest diameter at 90° to the line used to provide the numerator as the denominator. Box plots and other statistics were calculated using MS-Excel, together with the add-in template downloadable from http://www.vertex42.com/. In descriptive statistics, a box plot is a convenient way of graphically depicting groups of numerical data through their quartiles [72]. P values were calculated from the means, the numbers of objects measured in each class and the standard deviations using the Excel add-in available via http://www.talkstats.com/attachment.php?attachmentid=261&d=1213281245 and the facility at http://www.graphpad.com/quickcalcs/ttest1.cfm?Format=SD.

The cover slips with prepared smears were incubated at room temperature for 5 minutes and were then immersed in 0.075 M sodium phosphate buffer (pH 7.4) and finally placed on a shaker for 2 minutes. Smears were fixed in 2.5% gluteraldehyde/formaldehyde (1∶1) in PBS solution with a pH of 7.4 for 30 minutes, followed by rinsing 3× in phosphate buffer for five minutes before being fixed for 30 minutes with 1% osmium tetroxide (OsO_4_). The samples were again rinsed 3× with PBS for five minutes and were dehydrated serially in 30%, 50%, 70%, 90% and three times with 100% ethanol. The material was critical point dried, mounted and coated with carbon. A Zeiss ULTRA plus FEG-SEM with InLens capabilities were used to study the surface morphology of platelets and micrographs were taken 1 kV. This instrument is located in the Microscopy and Microanalysis Unit of the University of Pretoria, Pretoria, South Africa.

## Results

We choose to provide the data in the form of micrographs, that illustrate the typical morphologies we observe, along with statistical analyses of many measurements to provide the necessary robustness or powering [72]. All individuals were tested for the HH mutation. They were sent for testing by their medical practitioners due to the fact that the all had symptoms of iron overload including possible organ damage. Most of the HH individuals have increased SF levels, while all HF individuals (by definition) have increased SF levels. Increased serum iron was taken as above 30 µmol.L^−1^; transferrin levels that were below 2.1 g/L^−1^ were taken as atypical and % saturation above 45% were taken as atypical. Table 2 shows the reference serum ferritin values typically used to determine the presence of iron overload; while Table 3 shows normal ranges for serum iron, transferrin and % transferrin saturation.

**Table 2:** A series of analyses indicating that serum ferritin levels are taken as an indicator of Hemochromatosis, hyperferritinemia, and in healthy individuals.

Healthy individuals
Males: 25 –300 µg/L^−1^; Females: 25 – 200 µg/L^−1^ [73]
**Levels of serum ferritin taken as indication for Hemochromatosis**
Serum ferritin above approximately females 200 µg/L^−1^ and males 300 µg/L^−1^. [41], [53], [74]–[79].
88% of males with homozygous C282Y mutation individuals serum ferritin levels were greater than 300 µg/L^−1^ and in females 57% had levels greater than 200 µg/L^−1^ [77].
Severe overload - serum ferritin levels more than 1000 µg/L^−1^ [55], [80].
Severe overload - serum ferritin levels more than ≥239 µg/L^−1^ [51].
Ideal maintenance for Hemochromatosis: 25 – 50 µg/L^−1^ [73], [75].

**Table 3:** Normal values for serum iron, transferrin and % transferrin saturation.

Normal values for serum iron
11.6 – 31.4 µmol/L^−1^ [81]
Normal values for transferrin
2.2 – 3.7 g/L^−1^ [81]
Normal values for % transferrin saturation
20–50% [81]up to 45% [82]–[84]

**Figure 2:**
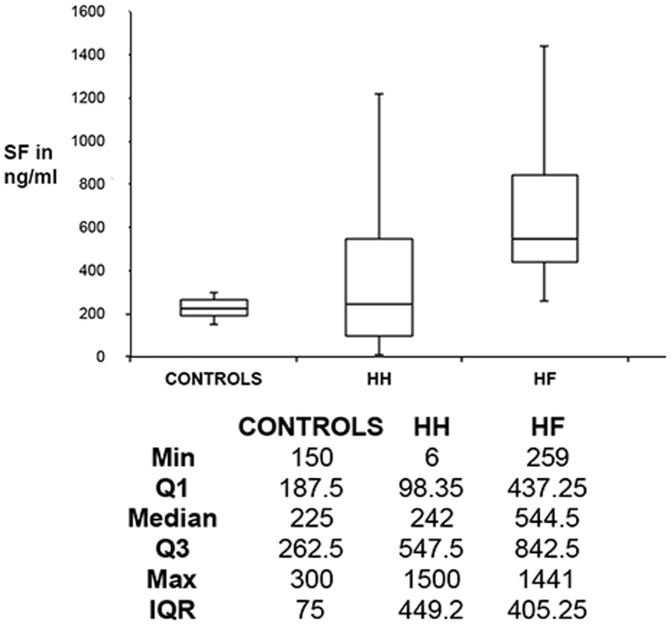
A box plot drawn from serum ferritin (SF) values for healthy individuals, HH and HF individuals.

**Figure 3:**
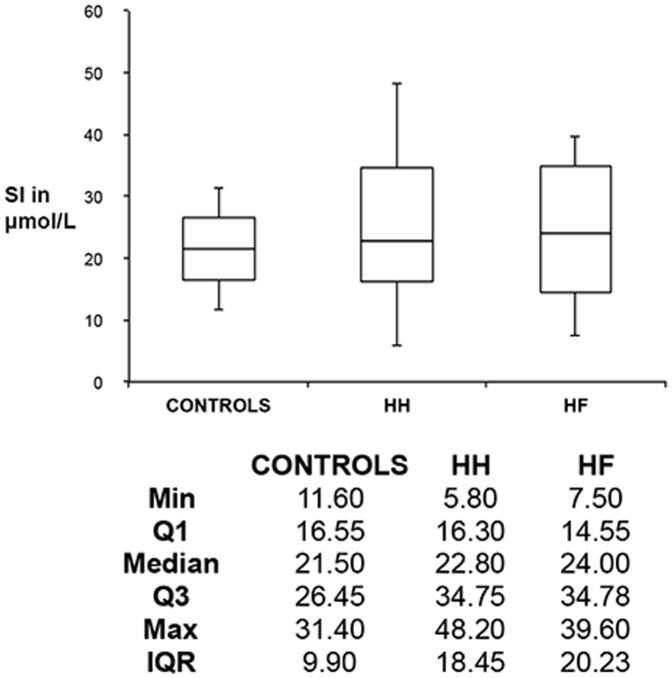
A box plot drawn from serum iron (SI) values for healthy individuals, HH and HF individuals.

Tables 4 and 5 show the profiles of our HH and HF patients, while Figures 2 and 3 show box plots and statistics for serum ferritins (SFs) and serum irons (SIs) for controls, HH and HF individuals. Statistical analyses (data shown at the bottom of Figures 2 and 3) confirm that both the HH and HF groups have increased SF levels, compared to those of healthy individuals (p-value<0.05). We could not find any correlation between serum iron, transferrin and % saturation and the presence of the HH mutation (an illustration of the serum iron data is given in Table 5). However, many patients with HF also had increased SF levels and in some cases the 3 other values were also increased (see Table 5). From the data in Table 5, it therefore seems as if SF levels are the iron-related value that most effectively imply or reflect the presence of the HH mutation.

**Table 4:** Profiles of Hereditary Hemochromatosis and genetically wild type individuals with high serum ferritin (SF) levels used in this study (rows in italics show individuals with SF values that are within normal ranges: females ≤200 ng/mL^−1^; males ≤300 ng/mL^−1^).

Gene	Serum ferritin (ng/mL^−1^)	Age	Gender	Representative Figure
H63D/H63D	1613	52	M	
*C282Y/C282Y*	*15*	*22*	*M*	*3B*
C282Y/C282Y	389	44	M	
C282Y/C282Y	1113	46	M	
C282Y/C282Y	508	37	F	3A
C282Y/H63D	374	72	F	
*C282Y/H63D*	*5*	*24*	*F*	
*C282Y/H63D*	*69*	*11*	*F*	
*C282Y/H63D*	*27*	*13*	*M*	
C282Y/H63D	1019	58	M	
C282Y/H63D	1166	59	F	3E
C282Y/wild type	1344	63	M	
C282Y/wild type	506	53	M	
*C282Y/wild type*	*79*	*12*	*M*	
*C282Y/wild type*	*68*	*64*	*M*	*4G, 5G, 6G, 7G*
C282Y/wild type	219	54	F	3D
H63D/wild type	625	52	M	4,5,6,7 A–F
H63D/wild type	468	48	M	
*H63D/wild type*	*179*	*19*	*M*	*3G*
H63D/wild type	594	46	M	
H63D/wild type	242	50	F	
H63D/wild type	634	44	F	4A, B, C
**AVERAGE**	**511.64**	**43**		
**WILD TYPE (Genetically tested due to a history of familial HH and high SF)**				
Wild type	242	57	F	
Wild type	841	55	F	
Wild type	506	53	M	3H
Wild type	303	51	M	3C
Wild type	1230	66	M	3F
Wild type	790	58	F	
Wild type	479	86	F	
Wild type	1736	62	M	
Wild type	860	41	M	
Wild type	453	57	M	
**AVERAGE**	**512**	**59**		

**Table 5:** HH and wild type individuals with age, gender, free iron, transferrin and % saturation levels.

Mutation	Serum ferritin ng/mL^−1^	Gender	Age	Serum Iron (µmol/L^−1^)	Transferrin (g/L^−1^)	% Saturation
C282Y/H63D	7	F	24	21.3	2.6	33
C282Y/H63D	1218	M	54	32.4	2	65
C282Y/H63D	9	M	64	5.8	3.5	7
C282Y/wild type	32.41	F	12	17.2	3.1	22
C282Y/wild type	313.8	M	12	37.3	2.1	71
C282Y/wild type	118	F	57	22.6	2.2	41
C282Y/wild type	113.7	F	39	25.8	2	52
C282Y/wild type	197	M	20	48.2	2.2	88
C282Y/wild type	807	M	48	22.8	2.7	34
C282Y/C282Y	389.7	M	44	21.5	2.1	41
C282Y/C282Y	501	M	26	40.3	2.1	77
H63D/H63D	83	F	43	10.8	2.1	21
H63D/wild type	634.1	F	44	29.2	3.4	34
H63D/wild type	242	F	50	9.7	2	19
H63D/wild type	159	M	63	13.3	3.4	16
H63D/wild type	6	F	44	13.2	2.8	19
H63D/wild type	594	M	46	34.8	3.4	41
H63D/wild type	469	M	39	15.4	2.4	25
H63D/wild type	483	M	56	40.1	2.7	59
H63D/wild type	1500	M	47	30.1	1.1	95
H63D/wild type	35	F	15	34.7	2.2	63
H63D/wild type	126	F	41	21.3	2.5	34
H63D/wild type	736	M	61	37.4	2.5	60
**AVERAGES**	**381.46**		**42**	**25.4**	**2.5**	**44**
Wild type	303	M	51	11.7	3.1	15
Wild type	790	F	58	28.8	2.9	40
Wild type	860	M	41	22.1	2.7	33
Wild type	453	M	57	23.3	2	47
Wild type	568	M	48	13.1	2	26
Wild type	527	M	51	24.7	2.2	45
Wild type	269	M	62	38.6	2.1	74
Wild type	1386	M	38	29.9	2.6	46
Wild type	1441	M	39	39.6	2.6	61
Wild type	259	F	63	9.8	2.7	15
Wild type	432	F	53	7.5	1.8	17
Wild type	562	M	44	18.9	2.6	29
Wild type	455	M	56	36.4	3.1	47
Wild type	1434	M	63	36.7	2.3	64
**AVERAGES**	**695.64**		**51.71**	**24.4**	**2.5**	**40**

HH and wild type individuals with serum ferritin (normal values: females ≤200 ng/mL^−1^; males ≤300 ng/mL^−1^) gender, age, serum iron (normal values: 11.6 – 31.4 µmol/L^−1^), transferrin (normal values: 2.2 – 3.7 g/L^−1^) and % saturation levels (normal values: 20 – 50%). Bold values indicate where levels do not fall into normal value.

**Figure 4:**
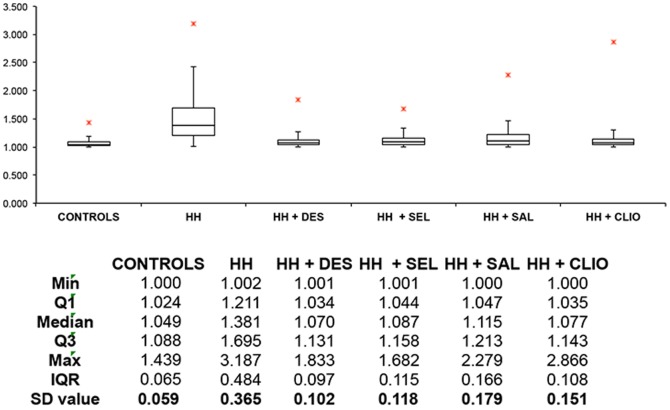
A box plot of axial ratios of 20 cells from 17 healthy individuals (n = 340) versus axial ratios of 20 cells from 13 HH individuals (n = 260) with and without chelating and other compounds (n = 260 per compound). Micrographs were taken at 100x magnification with a Nikon Optiphod transmitted light microscope (Nikon Instech Co., Kanagawa, Japan).

**Figure 5:**
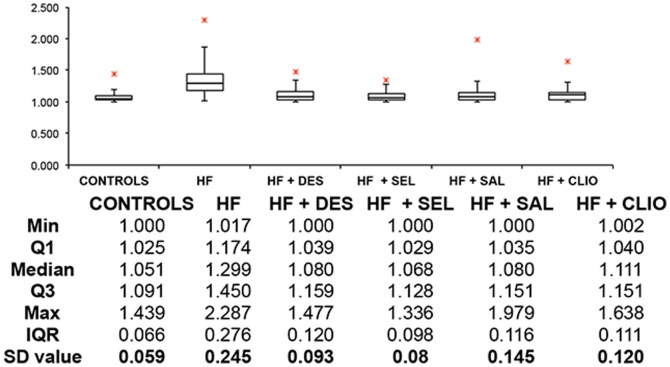
A box plot of axial ratios of 20 cells from 17 healthy individuals versus 20 cells from 4 HF individuals (n = 80) with and without chelating and other compounds (n = 80 per compound). Micrographs were taken at 100× magnification with a Nikon Optiphod transmitted light microscope (Nikon Instech Co., Kanagawa, Japan).

Figure 4 shows a box plot of axial ratios, with statistics for controls and HH individuals; and also HH RBCs with and without chelating and hydroxyl trapping agents. Figure 5 similarly shows a box plot of axial ratios for HF individuals, and also HF RBCs with and without chelating and hydroxyl trapping agents.

Axial ratios of RBCs in healthy control individuals are close to 1, which is indicative of their well-known, common discoid shape. Both the HH and wild type individuals show a significantly elongated shape as seen in their axial ratios, and these also have a much greater variance. However, when the iron-chelating compounds are added, RBCs appear to revert to the typical discoid shape. Although the serum ferritin concentrations in HH individuals are not significantly different from those of the controls, they do exhibit a very much greater variance, while the concentrations in those with hyperferritinaemia are of course (by definition) substantially greater (Figure 2). By contrast, there were no significant differences in serum iron (transferrin-bound iron) between the controls, HH or HF samples (Figure 3).

P values assess the probability of the null hypothesis being true (i.e. that all objects are from the same population and thus not ‘different’ from each other) [72]. For controls (17 individuals), HH (13 individuals) and HF (4 individuals), axial ratios of 20 cells per individual were measured (controls: n = 340; HH: n = 260; HF: n = 80). For each of these HH and HF individuals, compounds were added to WB and axial ratios of 20 cells per added compound were again measured. The mean values for the axial ratios for HH cells differed highly significantly from those of control cells (p<0.0001), and also from those treated with desferal, salicylate, selenite and clioquinol (P<0.0001 in every case). As with the HH, the axial ratios in HF patients also differed highly significantly from those of controls (P<0.0001) and from those treated with desferal, salicylate, selenite or clioquinol (P<0.0001 in every case).

**Figure 6:**
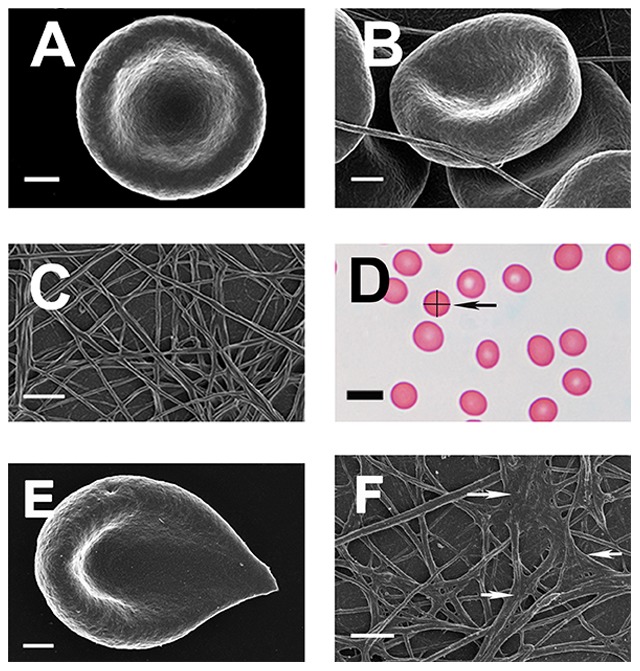
RBCs and fibrin networks from healthy individuals with SF levels within normal ranges. **A**) RBC **B**) RBC with added thrombin; **C**) Platelet rich plasma smear with added thrombin. **D**) Typical light microscopy smear from a healthy individual. The cell arrowed illustrates the means by which we determined the axial ratios. **E**) Healthy RBC exposed to physiological levels of iron. **F**) Healthy PRP exposed to physiological levels of iron showing matted masses (white arrows). All SEM micrographs scales  = 1 µm; Light microscopy scale  = 10 µm.

**Figure 7:**
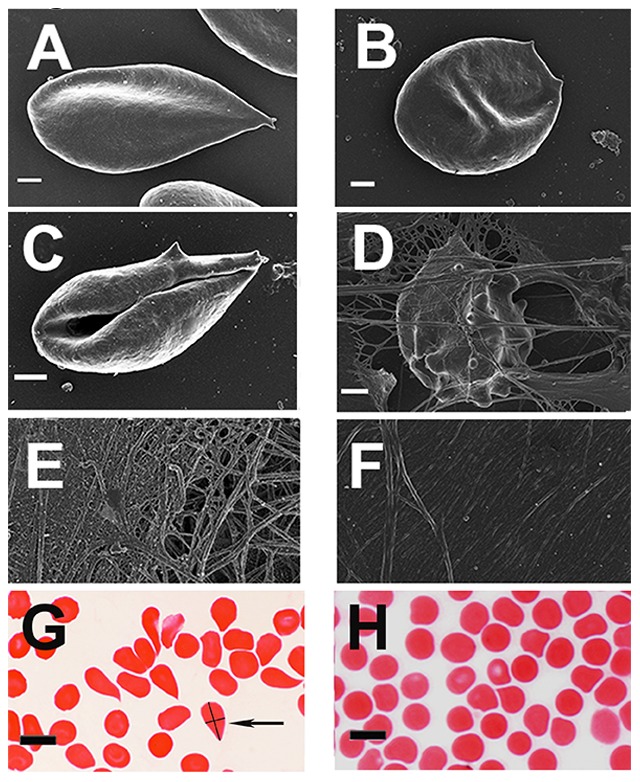
Micrographs from hereditary hemochromatosis (HH) and hyperferritinemia (HF) individuals. **A**) RBC from HH individual with high SF (508 µg/L^−1^) (C282Y/C282Y); **B**) RBC from HH individual with low SF (15 µg/L^−1^) due to regular phlebotomy (C282Y/wild type) **C**) Whole blood smear, showing elongated RBC from a HF individual with high SF (303 µg/L^−1^); **D**) Whole blood smear, from HH with added thrombin (C282Y/wild type) (219 µg/L^−1^); **E**) Platelet rich plasma smear from HH with added thrombin (C282Y/H63D) (1166 µg/L^−1^); **F**) Platelet rich plasma smear from HF individual with added thrombin (1230 µg/L^−1^); **G**) Light microscopy smear from a H63D/wild type individual (179 µg/L^−1^) - The cell arrowed illustrates the means by which we determined the axial ratios; **H**) Light microscopy smear from a HF individual with high iron levels (506 µg/L^−1^). All SEM micrographs scales  = 1 µm. Light microscopy micrographs scales  = 10 µm.

**Figure 8:**
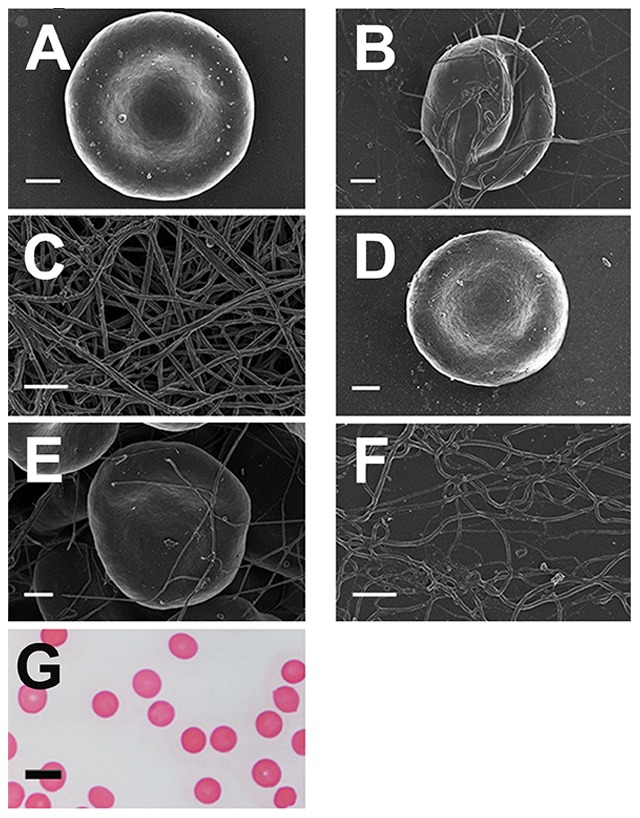
Micrographs of samples from patients with hereditary hemochromatosis with added desferal. **A**) Whole blood with added 10 mM desferal (H63D/wild type); **B**) Whole blood, with added thrombin and 10 mM desferal (H63D/wild type); **C**) Platelet rich plasma smear, with added thrombin and 10 mM desferal (H63D/wild type); **D**) Whole blood with added 0.5 mM desferal (H63D/wild type); **E**) Whole blood, with added thrombin and 0.5 mM desferal (H63D/wild type); **F**) Platelet rich plasma smear, with added thrombin and 0.5 mM desferal (H63D/wild type); **G**) Light microscopy of whole blood with 0.5mM desferal (C282Y/wild type). All SEM micrographs scales  = 1 µm; light microscopy micrograph scale  = 10 µm.

**Figure 9:**
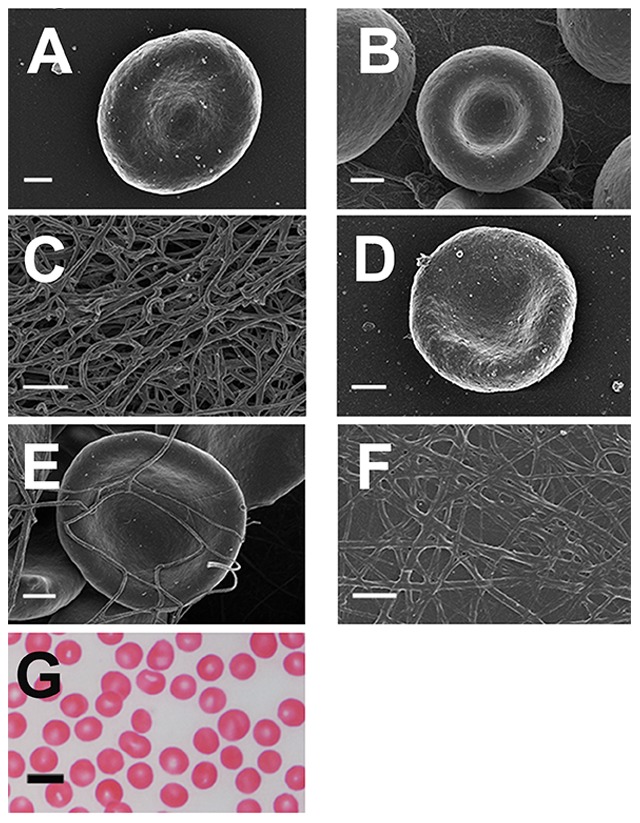
Micrographs of samples from patients with hereditary hemochromatosis with added sodium salicylate. **A**) Whole blood with added 10 mM sodium salicylate (H63D/wild type); **B**) Whole blood, with added thrombin and 10 mM sodium salicylate (H63D/wild type); **C**) Platelet rich plasma smear, with added thrombin and 10 mM sodium salicylate (H63D/wild type); **D**) Whole blood with added 0.5 mM sodium salicylate (H63D/wild type); **E**) Whole blood, with added thrombin and 0.5 mM sodium salicylate (H63D/wild type); **F**) Platelet rich plasma smear, with added thrombin and 0.5 mM sodium salicylate; **G**) Light microscopy of whole blood with 0.5 mM sodium salicylate (C282Y/wild type). All SEM micrographs scales  = 1 µm; light microscopy micrograph scale  = 10 µm.

**Figure 10:**
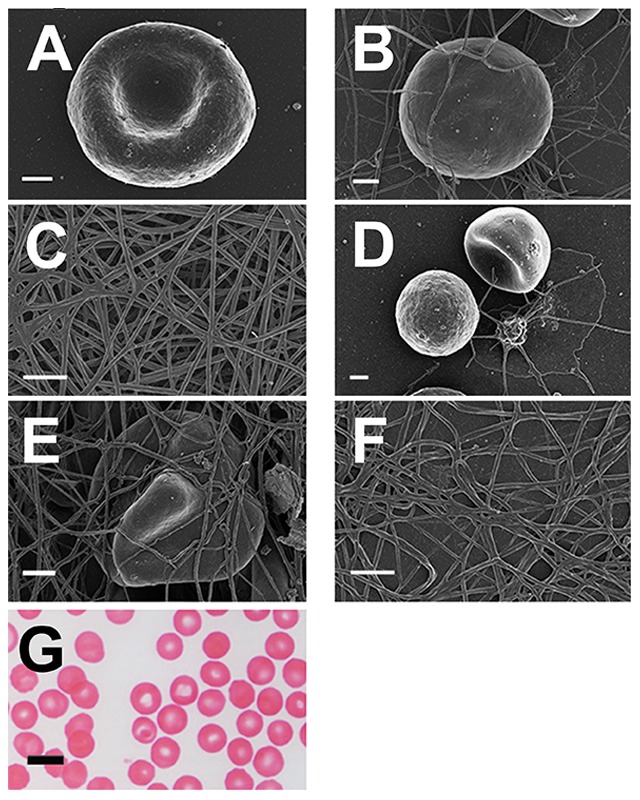
Micrographs of samples from patients with hereditary hemochromatosis with added sodium selenite; A) Whole blood with added 10 mM sodium selenite (H63D/wild type); B) Whole blood, with added thrombin and 10 mM sodium selenite (H63D/wild type); C) Platelet rich plasma smear, with added thrombin and 10 mM sodium selenite (H63D/wild type); D) Whole blood with added 0.5 mM sodium selenite (H63D/wild type); E) Whole blood, with added thrombin and 0.5 mM sodium selenite (H63D/wild type); F) Platelet rich plasma smear, with added thrombin and 0.5 mM sodium selenite (H63D/wild type); G) Light microscopy of whole blood with 0.5 mM sodium selenite (C282Y/wild type). All SEM micrographs scales  = 1 µm; light microscopy micrograph scale  = 10 µm.

**Figure 11:**
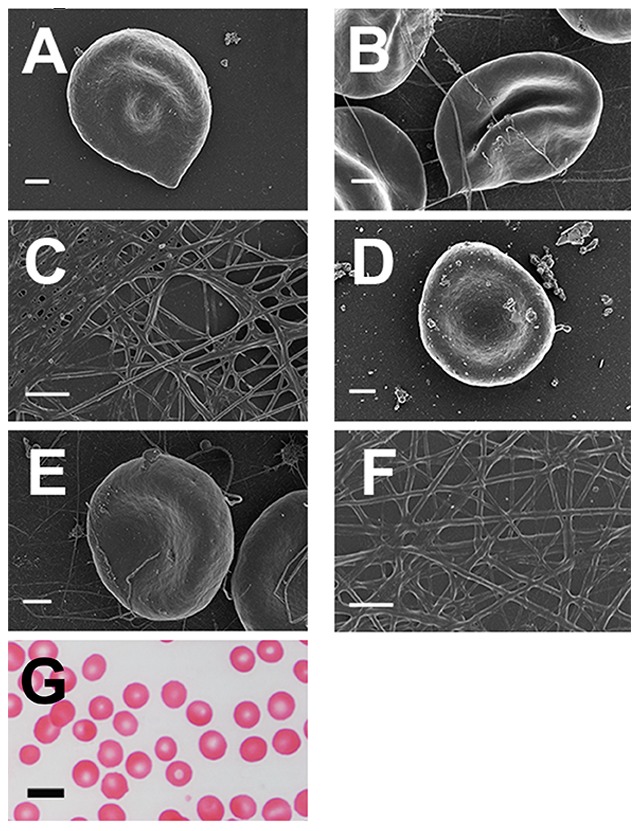
Micrographs of samples from patients with hereditary hemochromatosis with added clioquinol. **A**) Whole blood with added 10 mM clioquinol (H63D/wild type); **B**) Whole blood, with added thrombin and 10 mM clioquinol (H63D/wild type); **C**) Platelet rich plasma smear, with added thrombin and 10 mM clioquinol (H63D/wild type); **D**) Whole blood with added 0.5 mM clioquinol; **E**) Whole blood, with added thrombin and 0.5 mM clioquinol (H63D/wild type); **F**) Platelet rich plasma smear, with added thrombin and 0.5 mM clioquinol (H63D/wild type); **G**) Light microscopy of whole blood with 0.5 mM clioquinol (C282Y/wild type). All SEM micrographs scales  = 1 µm; light microscopy micrograph scale  = 10 µm.

Figures 6, 7, 8, 9, 10, 11 show SEM and LM micrographs (see the illustration in Figures 6D and 7G: RBC with measurement lines for axial ratios, indicated by arrows in each micrograph). Figure 6A–C shows SEM micrographs of typical healthy RBC, whole blood (WB) with thrombin, as well as PRP with added thrombin. These individuals do not have increased SF, and do not smoke nor use any chronic medication. As expected, RBCs of such healthy individuals show a typical discoid shape (Figure 6A). When thrombin is added to WB, the RBCs keep their typical discoid shape; however, fibrin fibers form over and around the RBCs (Figure 6B). When PRP is mixed with thrombin, a typical fiber net is formed (Figure 6C). Figure 6D shows a light microscopy smear of a healthy individual. We have previously shown that the exposure of whole blood to physiological levels of iron (0.03 mM FeCl_3_) causes RBC shape change [85]. This can be seen in Figure 3E. Figure 3F also shows how ‘healthy’ fibrin changes in the presence of iron.

By contrast, Figure 7A–D show SEM micrograph smears from blood taken from HH and HF individuals. HH RBCs typically have a substantially changed shape, where they become elongated, with pointed extensions (Figure 7A). Interestingly, in HH where SF is within normal ranges, RBCs still have an elongated shape (Figure 7B). This was noted in all individuals with the mutation but where SF levels are within the normal ranges. A changed RBC and fibrin network morphology was also noted for HF individuals with SF levels higher than 200 µg/L^−1^ (females) and 300 µg/L^−1^ (males) (Figure 7C). Thus either HH or HF alone is sufficient to cause the aberrant morphology. Figure 7D shows whole blood from a female HH individual (SF = 219 µg/L^−1^) with added thrombin, where the RBCs are entrapped and deformed in the fibrin mesh. Figure 7E shows PRP and thrombin from an HH individual (SF = 1166 µg/L^−1^), where the typical fibrin fibers coagulate to form a tighter meshed network. It is known that iron ions may inhibit thrombin activity ([86]). Here this changed fibrin morphology may in part be the result of this inhibition. The same ultrastructure is seen in HF individuals (Figure 7F – SF = 1230 µg/L^−1^).

Figure 7G shows a light microscopy micrograph of a typical HH individual with SF in the normal range (179 µg/L^−1^); and Figure 7H, that of an HF individual (SF = 506 µg/L^−1^). The shape changes seen in some of the RBCs are typical of a representative light microscopy view of the whole smear. Interestingly, all HH individuals whose SF levels are within normal ranges (Table 4, low SF levels shown in italics) still have a changed RBC shape.

We also exposed PRP and WB from HH and HF (with and without thrombin) individuals to 2.5 mM and 3.33 mM and desferal (Figure 8A–C), sodium salicylate (Figure 9A–C), sodium selenite (Figure 10A–C) or clioquinol (Figure 11A–C) (Table 1). A second round of experiments (Figures 8–11 D–F) was done at 20-fold lower concentration of the compounds were added (Table 1).

To assess the extent of the changes, RBC light microscopy was included for low concentrations of all compounds (Figures 8–11 G). Desferal is the classical iron chelator; however, it has poor gastrointestinal absorption and therefore has to be administered intravenously or subcutaneously [87]–[89]. Sodium salicylate is a known trap for free radicals [90], [91], and is an active metabolite of aspirin [92] and we previously showed that it has a protective effect on fibrin fiber networks after iron exposure [7]. Sodium selenite is an assimilable form of an essential trace element (Se) with antioxidant, immunological, and anti-inflammatory properties [93]. This said, we note that we cannot absolutely exclude that salicylate and selenite also have some iron-chelating activities, since catechol (structurally related to salicylate) does [94], and salicylate is part of the siderophore enterobactin [95], while the solubility product of ferric selenite is rather low [96], [97]. Clioquinol has the ability to chelate and redistribute iron [98] and is emerging as a potential therapy for some diseases, such as Alzheimer's disease [99] and cancer [100]. The ‘high’ concentrations of all the compounds stabilized the RBC and fibrin morphology. Although the lower concentrations also showed a stabilizing effect, it was not as profound as that seen with the high concentrations. Nevertheless, here we saw that both concentrations of the compounds stabilized ultrastructure in both HH (low and high SF levels) and HF individuals.

## Discussion

Excess iron levels are associated with Alzheimer's disease, Parkinson's disease, Huntington's disease, Friedreich's ataxia and other neurological disorders, cancer, Fanconi anemia, stroke, heart disease, diabetes and ageing [1], [2], [5], [18], [19], [54], [101]–[110]. One of the hereditary diseases necessarily associated with iron overload is hemochromatosis, where iron overload causes oxidative stress that ultimately damages organs. An unanswered question remains as to what extent, and by what mechanisms, hereditary (or other) iron overload conditions may contribute to the clinical manifestation of the conditions listed above.

We have recently shown that high added iron may impact on the coagulation profile and RBC ultrastructure [66], [111]. Previously, we have documented that ferric ions can activate non-enzymatic blood coagulation, resulting in the formation of fibrin-like dense matted deposits (DMDs) demonstrable by SEM [112]. Azizova and co-workers also noted that iron causes oxidative modification of thrombin [86]. RBCs also change morphology under elevated iron levels [96]. Further, it is also known that hemoglobin (Hb) content in HH is raised [113], [114]; however, the ultrastructure of fibrin fibers and RBCs in blood in HH seem not to have been investigated previously. Currently, we know that iron may change fibrin fiber shape and packaging, when added at physiological levels [8], [66]. We have also shown [8] that in diabetes, if elevated iron levels are present, changes in fibrin fibers as well as RBC structure occur. This was ascribed to another, pathological pathway of fibrin formation initiated by free iron (initially as Fe (III)), leading to the formation of highly reactive oxygen species such as the hydroxyl radical, that can oxidise and insolubilize proteins, a process that might be inhibited by iron-chelating compounds [8]. The final product of such a pathway is a fibrin-like material, termed dense matted deposits (DMDs) that are remarkably resistant to proteolytic degradation. We developed a laboratory platelet rich plasma (PRP) as well as a functional fibrinogen model where we used scanning electron microscopy (SEM) [58] to show that iron-chelating agents can be effective inhibitors of DMD formation [8]. Of a small range tested, the most active inhibitors of DMD formation proved to be desferal, clioquinol and curcumin, whereas epigallocatechin gallate and deferiprone were less effective. In the present work, we also investigated the protective effect of the direct free radical scavenger, sodium salicylate, as well as sodium selenite, by pretreating iron-exposed PRP and purified fibrinogen with these candidate molecules [7], though as noted above we cannot entirely exclude that they can chelate iron too. We suggested that the hydroxyl radicals produced by iron exposure, are neutralized e.g. by their conversion to molecular oxygen and water, thus inhibiting the formation of dense matted fibrin deposits in human blood and our laboratory fibrinogen model [7]. We note too the role of iron in the production of other dense cellular deposits such as lipofuscin (e.g. [115]–[118]), and we should also recognise that the ferric iron, as a trivalent cation, necessarily has profound electrostatic effects, simply from the Debye-Hückel theory. Finally, we note that that patients do have (excess) unliganded iron, that we also measure the variations in ferritin levels between individuals, and ferritin, even in serum, contains iron (see e.g. [119]–[121].

Perhaps surprisingly, very little is known about the RBC ultrastructure and fibrin network packaging in hemochromatosis individuals. In 1997 Akoev and co-workers showed that RBC membranes in hemochromatosis display an aggregation and enlargement of intra-membrane particles in comparison with structures seen in membranes from healthy donors [122]. In the present work, we demonstrate that the gross morphology of RBCs from HH individuals, as well as their fibrin fiber ultrastructure, is changed, where RBCs form pointed extensions and are distorted, with a much greater axial ratio compared to the appearance seen in the normal discoid samples. Previously, we have also shown with atomic force microscopy (AFM), that in diabetes the RBC membrane architecture is changed [123]. The RBC membrane consists of an overlaying asymmetric phospholipid bilayer membrane [124], supported by an underlying spectrin-actin cytoskeletal complex, which is interconnected by junctional complexes, resulting in a simple hexagonal geometric matrix. The associations between spectrin and actin with the junctional and ankyrin complexes are of fundamental importance for allowing erythrocytes to maintain their shape [125]. The plasma membrane is anchored to the spectrin network mainly by the protein ankyrin and the trans-membrane proteins band 3 (anion transport protein) and band 4.1 (55 kDa actin-binding protein) [126]– and is substantially responsible for controlling the rheological behavior and for withstanding the physical forces associated with circulatory transport.

We reported that in diabetes a decreased surface roughness is present, and that this is indicative of superficial protein structure rearrangement [123]. Given the effects of non-membrane-permeant chelators on the ability to reverse the morphological changes observed in the current study, we suggest that the change in RBC ultrastructure is driven by RBC membrane-induced architectural changes. We therefore agree with Akoev and coworkers that membrane architecture is changed in HH. This view is also consistent with the well-known ability of amphipathic cationic and anionic drugs to affect the membrane architecture of RBCs [67], [129]–[132].

In healthy individuals (with normal SF, free iron, transferrin and saturation levels), RBCs are typically discoid-shaped, even following the addition of thrombin (Figure 6A and B). The box plot analyses also reflect this (Figure 4 and 5). When the coagulation pathways are activated, fibrin fibers are generated (in the presence of thrombin), forming a clot. This clot has RBCs trapped in the network. This was also seen in our laboratory investigation (Figure 6B). In HH, as well as in HF individuals, the RBCs are highly entwined in the fibrin mesh, which might ultimately result in a tighter clot (only HH individual shown - Figure 7D).

Here we also show the effects of high and lower physiological level exposure of desferal, salicylate, sodium selenite or clioquinol. The higher additive concentrations (Figure 8–11 A–C) show a definite RBC and fibrin network stabilization as noted with the SEM data. Desferal stabilizes the RBC ultrastructure with and without thrombin, and fibrin fibers also appear more like those of a healthy individual (Figure 8). With the high desferal concentration, RBCs return to the typical, normal discoid-shaped, and with added thrombin, they regain their discoid shape (Figure 8A and B). The lower desferal concentration does not have such a profound stabilizing effect as the higher concentration, as most of the RBCs appear slightly elliptical rather than discoid. This is also seen in the light microscopy micrograph (Figure 8G). PRP with added thrombin, show more individual fibers between thicker homogenous fibrin. Therefore, the typical net does not completely form (Figure 8F) in the manner seen with the higher desferal concentration. These results are seen for both HH and HF individuals, suggesting that the reasons for the changed ultrastructure is primarily due to the high SF levels.

In whole blood smears without thrombin, but with added sodium salicylate, RBCs do not have the pointed extensions (Figure 9A–C). However, with added thrombin, most of the RBCs seem to be folded around the fibrin fibers, changing the typical discoid shape (Figure 9B). Fibrin fibers were comparable to those of healthy individuals (Figure 9C). Lower concentrations of the additives did stabilize the RBC as well as fibrin fiber morphology (Figure 9D – F). Light microscopy of whole blood with these lower concentrations also shows the stabilizing of the RBCs (Figure 9G). The same pattern was seen in the presence of sodium selenite (Figure 10A–C), except that it seems as if the RBC kept their shape better in the presence of the sodium selenite and thrombin. Clioquinol showed anomalous results, where RBC with and without thrombin kept their pointed appearance and the fibrin fibers also coagulated into DMDs with very few individual fibers visible.

The lower additive concentrations (Figure 8–11 D–F) for all products, show a less prominent stabilizing effect when viewed with SEM. However, light microscopy of samples in the presence of the lower concentrations clearly show that most of the RBCs have returned to the discoid shape. This was noted for HH as well as wild type/wild type individuals with higher than the accepted healthy serum iron levels (200 ng/mL^−1^ for females and 300 ng/mL^−1^ for males).

Individuals with hemochromatosis – as an ‘iron overload disease’ – are well known to have significantly more ‘iron’ in their bodies than do normal controls, and this is considered to contribute to the attendant gross pathologies of this syndrome. Phlebotomy and iron chelation are thus two therapies in common use [73], [80], [87]. Here we establish that accompaniments of this excessive iron in hemochromatosis whole blood are changes in both RBC morphology and in fibrin fibers, possibly due to hydroxyl radical formation or to the presence of excess iron itself. If hydroxyl radicals are involved, we suggest that they can cause non-enzymatic changes to fibrin in the presence of thrombin and a changed RBC ultrastructure, where the cells lose their discoid shape and are easily deformed when fibrin and DMDs are produced in the presence of thrombin. Ferric ions may also bind to the outer surface of the RBC directly [58]. As expected, the classical iron chelator, desferal, showed a stabilizing effect on both fibrin fiber and RBC ultrastructure. Clioquinol is known to chelate and redistribute iron [98], [99], [133]–[136]. However, in the current work, it showed the least potential to stabilize RBCs and fibrin. By contrast, salicylate and sodium selenite showed excellent stabilizing properties. Previously we have shown that sodium selenite inhibits fibrinogen polymerization, and suggested that this may occur by oxidation of hydroxyl radicals and the concomitant reduction of Se^4+^ to Se^2+^ [7]. Salicylate is also known to be a direct free radical scavenger and recently it has been shown that it affords protection against rotenone-induced oxidative stress and therefore has neuroprotective potential against OH^•^ radical damage [92], [137]. In the current study, sodium selenite and sodium salicylate plausibly also inhibited the hydroxyl radicals produced by the increased iron present in hemochromatosis, but as mentioned above may well also have bound or chelated some of the free iron.

The current research has shown that iron causes structural changes, but that selected additives cause a reverting of the structure; this suggests that the damage seen is indeed reversible. As discussed in the previous paragraphs, iron causes oxidative stress in cells. However, in the current manuscript we did not look at the specific markers that might cause oxidative damage, e.g. the presence of ROS in the RBCs. Some of the effects might be purely due to binding, e.g. via electrostatic effects. However, published research suggests that in the presence of high iron levels, RBCs and their precursors have more ROS than do their normal counterparts [138]. Furthermore, it has also been shown that chelators, including deferiprone, deferasirox and deferoxamine reduce the oxidative status of thalassaemic RBCs [138]. Further research, including the unravelling of the exact molecular mechanisms behind the shape changes would provide important insights into the treatment of iron overload diseases; however, tt is outwith the scope of this paper.

There is also discussion [139], [140] as to the utility or otherwise of using HH individuals as blood donors [141]. The present findings, indicating that the aberrant erythrocyte morphology is a property of individual cells (and not an ensemble in the thermodynamic sense [142]), suggest that care may need to be taken in the use of blood from HH donors. The reversibility of the aberrant morphologies of the RBC of HH donors under the conditions normally used in blood banks should therefore be checked.

Overall, we found remarkable changes in the morphology of RBCs in individuals with HH and SF, and showed that to an extent these can be reversed by chelators of unliganded iron and molecules that are known to stop their sequelae in terms of hydroxyl radical formation. An interesting observation is that even if SF levels are within normal ranges for the HH individuals, they still have a changed RBC and fibrin network ultrastructure. SF levels are therefore not the only parameter that changes ultrastructureAt all events, as illustrated by the independence of HH and HF, the ability to cause a raising of serum ‘iron’ is a systems property, reflecting the interplay between SF and all other aspects of the iron metabolic network. In HH individuals and wild type individuals where SF is high, a changed RBC shape is also noted, and the axial ratios reflect this. We could not find a clear correlation between the 3 other typical pathology laboratory results requested by medical practitioners and the presence of the HH mutation (Table 5).

This said, it seems as if increased serum ferritin levels in the HF individuals do indeed cause (or accompany) changes in ultrastructure. This could be seen as consistent with the view that the morphological changes are caused not only by the raised Hb levels in such RBCs [113], [114] but by unliganded iron itself. Whether this aberrant morphology contributes to disease pathology is not known, but an interesting parallel can be made with sickle cell disease. Here it is definitely known that the altered RBC morphology contributes to pathology as the deformed erythrocytes struggle to pass through blood capillaries, often leading to stroke [143]–[148]. Iron parameters are often raised in sickle cell disease too, including as a result of transfusion treatment [149]–[155]. It would thus be of interest to assess the effects of iron chelators on sickle cell morphologies directly.
